# Laterality enhances numerical skills in the guppy, *Poecilia reticulata*

**DOI:** 10.3389/fnbeh.2015.00285

**Published:** 2015-10-26

**Authors:** Marco Dadda, Christian Agrillo, Angelo Bisazza, Culum Brown

**Affiliations:** ^1^Department of General Psychology, University of PadovaPadova, Italy; ^2^Centro di Neuroscienze Cognitive, University of PadovaPadova, Italy; ^3^Department of Biological Sciences, Macquarie UniversitySydney, NSW, Australia

**Keywords:** numerical discrimination, functional asymmetries, fishes, operant conditioning, quantity discrimination

## Abstract

It has been hypothesized that cerebral lateralization can significantly enhance cognition and that this was one of the primary selective forces shaping its wide-spread evolution amongst vertebrate taxa. Here, we tested this hypothesis by examining the link between cerebral lateralization and numerical discrimination. Guppies, *Poecilia reticulata*, were sorted into left, right and non-lateralized groups using a standard mirror test and their numerical discrimination abilities tested in both natural shoal choice and abstract contexts. Our results show that strongly lateralized guppies have enhanced numerical abilities compared to non-lateralized guppies irrespective of context. These data provide further credence to the notion that cerebral lateralization can enhance cognitive efficiency.

## Introduction

Cerebral lateralization refers to the partitioning of information processing in either hemisphere of the brain and is often overtly expressed as hand, eye or side preferences. Its ubiquity amongst vertebrate taxa suggests it has deep evolutionary origins and likely has multiple fitness benefits. It has been argued that enhanced processing capability was one of the driving forces lateralization evolution (Rogers, 2002). The potential mechanisms include the ability to process multiple sources of information simultaneously and reduced inter-hemispherical conflict, both of which should increase cognitive efficiency. To date, few researchers have studied the potential cognitive benefits of lateralization, most of these studies have identified enhanced cognitive abilities across a number of contexts. For example, individuals that are strongly lateralized were better able to forage under the threat of predation (Rogers et al., 2004; Dadda and Bisazza, 2006). Similarly strongly lateralized parrots were more competent at grain-pebble discrimination and were more likely to solve a string pull problem (Magat and Brown, 2009).

The fact that variation in the strength and direction of laterality exists both within and between species has led to the suggestion that there may also be costs associated with laterality. If there were no such costs, presumably all individuals would be strongly lateralized given the apparent cognitive benefits (i.e., the trait would run to fixation). Parrots, for example, vary greatly in the use of their feet when manipulating objects and this is tied to variation in feeding ecology (Brown and Magat, 2011). Indeed there is supporting evidence that enhanced laterality can have costs depending on the context providing a source of balancing selection. For example, strongly lateralized fishes took longer to solve a radial maze than weakly lateralized fishes because they had a tendency to repeatedly turn in a given direction rather than proceeding directly to the reward arm (Brown and Braithwaite, 2004). Strongly lateralized individuals also perform poorly when they are required to match information from two hemispheres simultaneously, for example, when fish view two different sized shoals with each eye (Dadda et al., 2009).

Lateralization of numerical abilities is receiving increasing attention in the literature. In recent years numerical abilities have been widely studied in animals (Beran, 2006; Cantlon and Brannon, 2007; Agrillo et al., 2010). It has been argued that both animals and humans share similar basic mechanisms for keeping track of objects and estimating relative abundance (Feigenson et al., 2004; Beran, 2008; Agrillo et al., 2012) which should not be surprising given the fitness advantages of doing so. There are numerous examples where numerical abilities are vital in the everyday lives of animals; estimating and comparing prey abundance in contrasting food patches (Hunt et al., 2008; Panteleeva et al., 2013), selecting to join the larger of two groups when threatened with predation (Buckingham et al., 2007; Gómez-Laplaza and Gerlai, 2011a), or electing to escalate fights based on numerical dominance (McComb et al., 1994; Benson-Amram et al., 2011). It seems likely that the ability to discriminate between sets of objects that are closely matched numerically is limited by cognitive capacity. Many animals struggle to differentiate between 3 vs. 4 or 4 vs. 5 (Hauser et al., 2000; Ward and Smuts, 2007; Hunt et al., 2008; Agrillo et al., 2012). Studies examining numerical discrimination have found inter-individual difference in numerical acuity (Beran, 2001; Halberda et al., 2008). For instance, in guppies, all subjects proved able to solve relatively easy discrimination tasks, such as 2 vs. 4 and 2 vs. 3, while only a few individuals were able to discriminate 3 vs. 4 and 4 vs. 5 (Bisazza et al., 2014a). Thus comparing sets of objects where the relative difference between sets diminishes but the absolute difference remains the same presents itself as an ideal task for testing cognitive ability.

There is evidence that some numerical abilities are lateralized in humans (Arsalidou and Taylor, 2011; Chassy and Grodd, 2012), but there are very few studies in non-human animals (dolphins; Kilian et al., 2005). Fish provide an excellent opportunity to examine the evolutionary origins of this apparent link. Moreover, variation in the strength of laterality may provide a functional explanation for individual variation in numerical discrimination in vertebrate taxa. Here, we examined the proposed cognitive benefits of laterality by investigating the link between the strength of cerebral lateralization and numerical ability using female guppies as our model system. Specifically we hypothesized that more strongly lateralized fish should be better at discriminating between two sets of objects than weakly lateralized fish. We took two approaches to address this problem. The first approach relied on the fish’s natural inclination to join the larger of two shoals when presented with a choice. The second approach required the fish to discriminate between sets of objects in a far more abstract context (orange circles on a white background). This second approach allowed us to control for a wide range of potentially confounding non-numerical factors, such as movement, density and surface area.

## Materials and Methods

### Subjects

We used adult female guppies, *Poecila reticulata* from an outbred domestic strain maintained at Dipartimento di Psicologia Generale (Università di Padova) that originated from about 200 individuals bought from a local pet shop. Female guppies are highly social and commonly form shoals of various sizes in their natural environment (Miletto Petrazzini et al., 2012). Fish were maintained in small heterosexual groups, fed twice a day and kept in 70-L glass aquaria with abundant vegetation and artificial lighting for 16 h per day. Water temperature was maintained at 25°C.

### Procedure

The subjects underwent three consecutive tests; mirror test, shoal choice, numerical discrimination training. On the first day, the fish underwent the mirror test to measure their laterality and were immediately transferred to the shoal choice test apparatus to measure their ability to discriminate shoal numerosity. Fish were then individually housed for 3 days. Fish housing consisted of a 35 × 50 × 32 cm plastic tank internally divided into 12 cells (11 × 12 × 30 cm each) by means of transparent panes. On Day 4, the subjects that met the laterality criterion started the numerical discrimination training.

### Mirror Test

**Figure 1:**
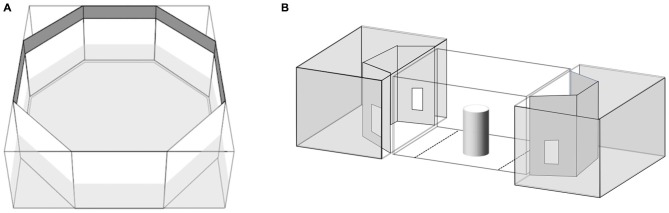
**(A)** Apparatus used for the mirror test. **(B)** Apparatus used for the shoal choice test. The central compartment housed the test subject while the adjacent aquaria housed the stimulus shoals. Choice zones are illustrated by the dotted lines on the floor of the central compartment.

Seventy subjects were observed in this test. We employed the same apparatus described elsewhere (Dadda et al., 2012; Figure 1A). Briefly, the fish was placed in a clear cylinder in the center of an octagonal tank with mirrored walls. After 2 min settling time the cylinder was raised and the time the subject spent shoaling with the virtual companion (a mirror image equates to an unfamiliar fish) on left or right was recorded for 5 min (De Santi et al., 2001). When placed in a novel tank containing a mirror, fish show a strong tendency to swim tightly parallel to the mirror (Dadda et al., 2007).

Recently a number of studies have questioned whether mirror-elicited behavior is effectively comparable to the same behavioral responses observed in real interactions. In particular brain gene expression, hormonal responses and aggression levels elicited by mirror images differ from those elicited by live stimuli (Oliveira et al., 2005; Desjardins and Fernald, 2010; Balzarini et al., 2014). It is worth noting that all of these studies refer to aggressive interactions between individuals whereas in the present study we employed the mirror test in a social context. *P. reticulata* is a strongly schooling species and the mirror test has been widely used in fish to measure social cooperation (De Santi et al., 2001; Dadda et al., 2007; Karenina et al., 2013; Regolin et al., 2013; Funghi et al., 2015).

Video recordings were analyzed using a computer program (Ciclic Timer Version 1.3). We considered the observations in which the fish was swimming along a mirror within 1 cm of the mirror. Observations made while the fish was perpendicular to the mirror were not considered. Only the subjects that swam at a maximum distance of 1 cm from the mirror for at least 70% of time during test were included.

We used the following formula to compute the laterality index: time spent swimming counter clockwise/(time spent swimming counter clockwise + time spent swimming clockwise). On the basis of the laterality index, individuals with a value >0.75 were classified as right-eye preference (from now on RE), individuals with a value <0.25 were classified as left-eye preference (from now on LE) and individuals with a value between 0.45 and 0.55 were classified as non-lateralized (from now on NL).

### Shoal Choice

Thirty-one subjects (9 RE, 9 LE and 13 NL) were observed in this test. We employed a modification of the apparatus described elsewhere (Agrillo et al., 2008). Briefly, the experimental apparatus was composed of three adjacent tanks (Figure 1B). The central one, the “subject tank”, housed the test fish (36 × 60 × 35 cm). A “stimulus tank” (36 × 60 × 35 cm) was placed at either end and faced the subject tank. A video camera was suspended 1 m above the subject tank and used to record the position of the subject during the tests. The subject tank was connected to an external tank (140 × 90 × 110 cm) that housed a large heterosexual group (approximately 80 individuals) of *P. reticulata* via a pipe (diameter 2 cm) in the center of the floor. Two pumps inserted into the external tank were connected to the subject tank allowing a continuous flow of water between them; this was done in order to control for the possibility that chemical cues released by frightened fishes affected the behavior of fishes taking part in the subsequent trials (Mirza and Chivers, 2002) and to maintain a familiar chemosensory environment.

Fishes used as stimuli were maintained throughout the test in the two stimulus tanks. These tanks were divided into two sectors by green plastic partitions; two doors allow passage from one sector to another and were closed 10 min before observations. A total of 28 adult females (14 for each tank) were used as stimuli. The stimulus tanks were lit by two fluorescent lamp (18 W) with water maintained at a temperature of 25° ± 2°C.

Subjects were introduced into a hollow transparent cylinder (8 cm diameter) connected to a pulley placed into the middle of the tank for 2 min. When the cylinder was raised, the subject’s position was recorded for 30 min. We used two different groups as stimuli, a larger group of 6 females and a smaller group of 4. In half of the tests the larger group was on the left and in the other half it was on the right.

From the video recordings we calculated the time spent by the subject female shoaling within a distance of 11 cm from the glass facing the stimulus tank (choice area; Agrillo et al., 2008; Piffer et al., 2012). The dependent variable was the proportion of time spent in the choice area close to the larger shoal. Subjects that spent less than the 40% of time within the choice area or did not visit both choice areas at least twice during the test were discarded. Two fish (both NL) failed to reach the criteria and were discarded from the analysis.

### Numerical Discrimination Training

**Figure 2:**
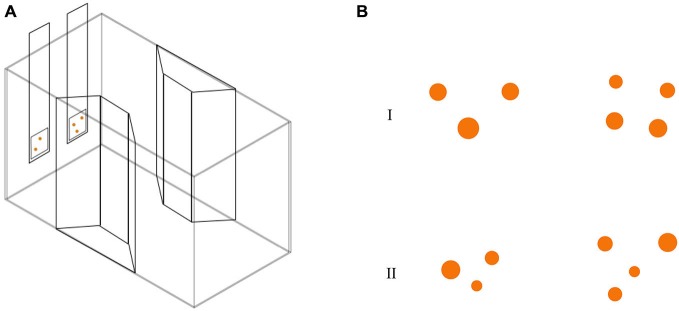
**(A)** Test apparatus for the abstract numerical discrimination test. Subjects were required to choose the stimulus with the greatest number of orange dots. **(B)** Stimuli used in numerical discrimination training consisted in two groups of dots differing in numerosity. Here, we depicted two examples of 3 vs. 4 contrast, with cumulative surface area controlled (I) and not controlled for (II).

Thirty-one subjects (9 RE, 9 LE and 13 NL) were observed in this test. We employed a modification of the apparatus described in a recent study (Agrillo et al., 2014). The experimental apparatus was composed of a glass tank (50 × 19 × 32 cm; Figure 2A) with a gravel substrate and water depth of 24 cm. The long walls were covered with green plastic material. Two transparent plastic sheets bended in a trapezoid-shape were placed in the middle of the tank providing an area in which two mirrors (29 × 5 cm) and live plants were placed. In order to reduce the potential effects of social isolation (Miletto Petrazzini et al., 2012) four juvenile fish were kept in the trapezoid-shape area of the tank. Eight identical experimental tanks were used and lit by two neon lights (36 W). Tanks were separated by means of green plastic partitions (50 × 32 cm).

Stimuli (Figure 2B) were inserted in a 5 × 5 cm square and were presented at the bottom of a 6 × 32 cm transparent plexiglass panel. Stimuli consisted of groups of orange circles differing in size on a white background. We used orange dots instead of black ones (more commonly adopted in the literature, see Piffer et al., 2013; Bisazza et al., 2014b) because a preliminary test showed that, with the present paradigm, guppies exhibited more interest for orange stimuli, probably because the food flakes here used as reward were orange as well. We presented different numerical contrasts: 2 vs. 3; 3 vs. 4 and 4 vs. 5. Stimuli selected for the experiment were extracted from a pool of 24 different pairs for each numerical contrast. Both the size and position of the circles were changed across sets since numerosity can co-vary with other physical attributes of the stimuli, such as area, brightness and density of the items. This non-numerical information—commonly called “continuous quantities” can be used by animals instead of numerical information to select the larger/smaller group (Pisa and Agrillo, 2009; Krusche et al., 2010). Stimuli were controlled for continuous quantities using some of the procedures commonly adopted in the study of numerical abilities of non-human animals (Cantlon and Brannon, 2007; Beran, 2008; Agrillo et al., 2014). In particular cumulative surface area was controlled for half of the stimuli. However, as a by-product of controlling for cumulative surface area, smaller than average figures were more frequent in the larger group. As a consequence, half of the stimuli were not controlled for cumulative surface area (e.g., number and area were simultaneously available). In addition, as density (inter-item distance) and the overall space encompassed by the most lateral figures are inversely related, half of the stimuli were controlled for the density of the items, half of the stimuli were controlled for the overall space occupied by the two arrays.

The experiment lasted for 13 days and was divided into two phases: (1) training (days 1–4) and (2) test (days 5–13). The aim of the training phase was to permit fish to familiarize themselves with the experimental apparatus and with the plexiglass panels. On days 1–2 fish were fed with commercial food flakes (GVG Sera) twice a day. On days 3–4 fish were fed six times a day. Three small pieces of food flakes (2 × 2 mm approx.) were attached to the stimuli in correspondence to each circle. A single panel was inserted on the short wall and kept in the tank until the fish ate the food flakes. The position (front/back) of presentation was counterbalanced over trials. At the end of the fourth day the fish were captured and placed into the area delimited by the plastic sheets (two for each area).

The test phase consisted of three different numerical contrasts (2 vs. 3, 3 vs. 4 and 4 vs. 5). Fish were reinforced to select the larger numerosities. On days 5–7 (2 vs. 3) fish received eight trials per day (for a total of 24 trials). During each trials two stimuli panels were inserted simultaneously and attached on the short walls. A small piece (2 × 2 mm) of food flake (one for each panels) was attached 4 cm above the stimulus. As soon as the subjects touched the reinforced stimulus with its snout, the corresponding panel was lowered to release the food flake while the other panel was removed from the tank. As a measure of their capacity to discriminate the two numerosities, we considered the first stimulus touched by the subject. On days 5–6 the two panels were kept in the tank until the subject touched the larger stimulus whereas on day 7 as soon as the fish touched the smaller stimulus both panels were removed from the tank without any reinforcement. We counterbalanced the position of the stimuli (left-right), the position of presentation (front/back) and the set of stimuli (controlled for cumulative surface area/non controlled) over trials.

On days 8–10 fish were tested with the 3 vs. 4 and on days 11–13 with the 4 vs. 5 numerical contrasts following the same procedure described above but for these contrasts subjects were reinforced only if their first choice was toward the largest stimulus. As dependent variable we considered the proportion of correct choices.

### Statistics

In the mirror test, shoal choice test and numerical discrimination training test departures from random choices (50%) were estimated by one-sample two-tailed *t*-tests performed respectively on the mean values of the laterality index, on the proportion of time spent close to the largest shoal and on the proportion of correct choices. Kurtosis was estimated assuming that a normal distribution has kurtosis 0 where any deviation from 0 is indicative of a non-Gaussian distribution (Snedecor and Cochran, 1989). Differences across groups were estimated by the analysis of variance (ANOVA) following checking of assumptions of normality and homogeneity of variance. All proportional data were arcsine square root transformed before analyses.

### Animal Ethics

The experiments comply with all laws of the country (Italy) in which they were performed (D.M. 116192) and the study was approved by the “Ministero della Salute” (permit number: 6726-2011). The methods were carried out in accordance with the approved guidelines.

## Results

### Mirror Test

**Figure 3:**
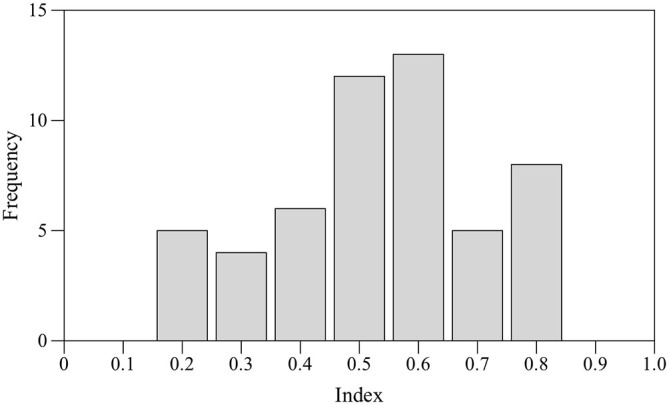
**Frequency distribution of the laterality index scores in the mirror test**.

Seventeen subjects (24%) did not shoal with their own mirror image for at least 70% of time and were discarded. Preference for right eye use was estimated by one sample two-tailed *t*-test performed on the mean values. No statistically significant bias for the right eye was found (*t*-test *t*_(52)_ = 0.989, *p* = 0.327). According to the laterality index adopted for this test a total of nine subjects were classified as RE, nine subjects were classified as LE and 13 were classified as NL. Score distribution appeared to be platykurtic (kurtosis *β*2 = −0.700; Figure 3).

### Shoal Choice

Preference for the larger shoal was estimated by one sample two-tailed *t*-test. Subjects from the three groups pooled together showed a significant choice of the larger shoal (mean ± SD 0.57 ± 0.09 *t*_(28)_ = 4.020, *p* < 0.001). When the three groups of fish were considered separately LE and RE subjects showed a significant choice of the larger shoal (*t*_(8)_ = 4.515, *p* = 0.002 and *t*_(8)_ = 2.711, *p* = 0.027 respectively) whereas NL subjects did not (*t*_(10)_ = 0.995, *p* = 0.343).

**Figure 4:**
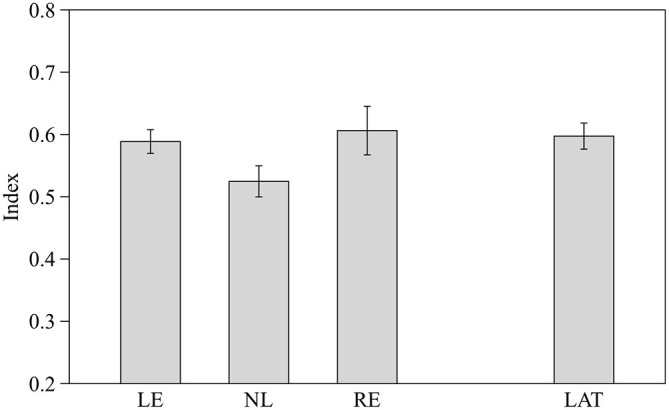
**Proportion of time spent in the in the choice area close to the largest shoal (6 vs. 4).** Means ± standard errors are reported. LE, fish favoring the left eye in the mirror test; NL, fish favoring neither eye in the mirror test; RE, fish favoring the right eye in the mirror test; and LAT, fish favoring the left or the right eye in the mirror test.

One-way ANOVA revealed no significant difference among the three groups of fish (*F*_2,26_ = 2.362, *p* = 0.114; Figure 4). However when LE and RE fish were considered as a single group of lateralized fish, one-way ANOVA showed a significant difference between lateralized and NL fish (*F*_1,27_ = 4.654, *p* = 0.040; Figure 4). RE and LE fish differ significantly when the position (right or left) of the largest shoal was considered (RE showed a preference for the right side where LE for the left side, *F*_1,27_ = 4.654, *p* = 0.040).

### Numerical Discrimination Training

Subjects from the three groups pooled together were able to discriminate 2 vs. 3 and 3 vs. 4 items (0.59 ± 0.07 *t*_(30)_ = 6.678, *p* < 0.001 and 0.57 ± 0.09 *t*_(30)_ = 3.866, *p* = 0.001 respectively) but not 4 vs. 5 items (0.51 ± 0.09 *t*_(30)_ = 0.827, *p* = 0.415).

**Figure 5:**
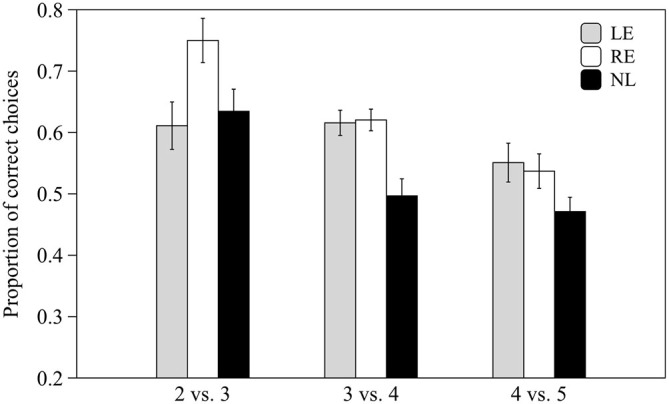
**Proportion of correct choices for the 2 vs. 3, 3 vs. 4 and 4 vs. 5 numerical contrasts.** Means ± standard errors are reported. LE, fish favoring the left eye in the mirror test; NL, fish favoring neither eye in the mirror test; RE, fish favoring the right eye in the mirror test.

When the three groups were considered separately, LE and RE subjects were able to discriminate both 2 vs. 3 and 3 vs. 4 items whereas NL subjects only discriminated the easiest contrast (see Table 1; Figure 5).

**Table 1:** **Performance of the three groups in the numerical contrasts considered**.

Group	2 vs. 3	3 vs. 4	4 vs. 5
LE	*t*_(8)_ = 2.861, *p* = 0.021	*t*_(8)_ = 5.521, *p* = 0.001	*t*_(8)_ = 1.619, *p* = 0.144
NL	*t*_(12)_ = 3.648, *p* = 0.003	*t*_(12)_ = −0.088, *p* = 0.931	*t*_(8)_ = 1.226, *p* = 0.244
RE	*t*_(8)_ = 6.301, *p* < 0.001	*t*_(8)_ = 6.704, *p* < 0.001	*t*_(8)_ = 1.334, *p* = 0.219

*LE, fish favoring the left eye in the mirror test; NL, fish favoring neither eye in the mirror test; RE, fish favoring the right eye in the mirror test*.

We calculated a comprehensive index of success considering the proportion of correct choices over the total number of trials for the three different numerical contrasts as follow: (total number of correct choices in the 2 vs. 3, 3 vs. 4 and 4 vs. 5 contrasts/total number of trials). Subjects from the three groups pooled together proved able to discriminate the larger stimulus (0.56 ± 0.08 *t*_(30)_ = 2.656, *p* = 0.013). When considered separately LE and RE subjects were able to discriminate the larger stimulus (*t*_(8)_ = 3.662, *p* = 0.006 and *t*_(8)_ = 4.754, *p* = 0.001 respectively) whereas NL subjects did not (*t*_(12)_ = 0.651, *p* = 0.527).

**Figure 6:**
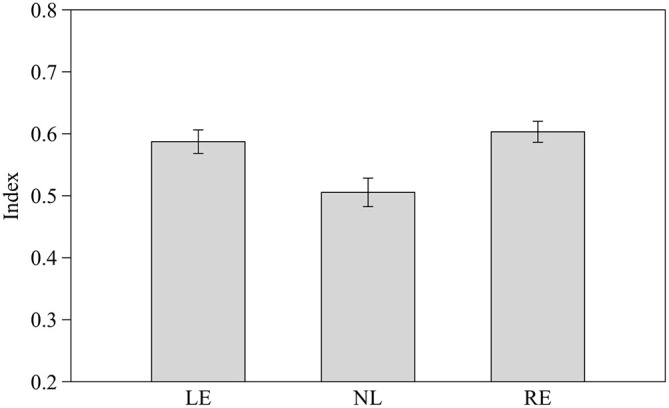
**Performance of the three groups considering the index of success in the three different numerical contrasts.** Means ± standard errors are reported. LE, fish favoring the left eye in the mirror test; NL, fish favoring neither eye in the mirror test; RE, fish favoring the right eye in the mirror test.

One-way ANOVA showed that the three groups differ significantly in their performance (*F*_2,28_ = 6.758, *p* = 0.004; Figure 6) and *post hoc* analysis (Scheffé method) reveals that NL subjects differ significantly from both LE (*p* = 0.014) and RE (*p* = 0.020) subjects.

We performed a Pearson correlation analysis considering the absolute index of laterality measured in the mirror test (0,5 |laterality index|), which provides a measure of the degree of lateralization independently from its direction, and the performance in both the shoal choice test and the numerical discrimination training test. Results showed that strongly lateralized fish performed better than poorly lateralized in both tests (*r* = 0.37, *p* = 0.048 and *r* = 0.585, *p* = 0.001 respectively for the shoal choice test and the numerical discrimination training test).

## Discussion

Overall our results strongly support the notion that enhanced cerebral lateralization increases cognitive ability. Both strongly left and right lateralized fish had enhanced numerical skills in comparison to non-lateralized fish across both a natural and an abstract task. In an ecologically relevant test, were fish were required to join the larger of two shoals, non-lateralized fish were incapable of discriminating between a shoal of 4 vs. 6 conspecifics. Strongly left and right biased fish, in contrast, readily chose the larger of the two shoals. However, in this test, numerosity co-varied with continuous quantities, such as overall volume of the conspecifics or density of conspecifics, hence no firm conclusion could be drawn on the exact quantitative mechanism used by guppies. It is worth noting, however, that in the real world there may be any number of cues that animals could use to differentiate between groups of objects that are not directly related to numerical abilities. For example, fish might discriminate the larger/smaller group by assessing the relative level of activity they see (or some crude sense collective of movement), the total surface area of the objects, or the relative density. Zebrafish, for example, choose the most active shoal which was experimentally manipulated simply by increasing water temperature (Pritchard et al., 2001). Similarly, Krusche et al. (2010) found that salamanders may chose prey patches based on the level of prey activity rather than actually enumerating them.

In a more abstract context, the numerical discrimination training test, We controlled for continuous quantities and this enabled us to directly assess whether lateralized and non-lateralized fish differed in the specific ability to use numerical information. The results of this task mirrored those in the shoal choice tests, with strongly lateralized fish showing better performance than non-lateralized fish. Lateralized fish could discriminate between 3 vs. 4 dots, but non-lateralized fish could not. This suggests that the difference in quantity discrimination ability between lateralized and non-lateralized guppies involves number processing. Moreover, left and right biased fish did not differ in their numerical abilities which is in agreement with other studies examining the link between cognition and laterality (Magat and Brown, 2009). These results suggest that it is the strength rather than the direction of laterality that is important in bolstering cognitive competency. Collectively these results show that strongly lateralized individuals have superior quantitative skills relative to non-lateralized individuals as we hypothesized.

It is worth noting that numerical contrasts presented in the discrimination training test can be included in what is commonly called the “subitizing range” in the human literature. Indeed several studies report the existence of two distinct systems of non-symbolic numerical processing of infants, children and adult humans: a precise system up to 3–4 units (subitizing) and an approximate number system (ANS) for larger numbers (e.g., Feigenson et al., 2004; Revkin et al., 2008). However, the existence of a subitizing-like system in non-human animals is contested, and most of the current studies are more in line with the idea of a single ANS for the whole numerical range. The data from fish experiments, however are equivocal (see Gómez-Laplaza and Gerlai, 2011a,b; Agrillo et al., 2012; Stancher et al., 2013; Miletto Petrazzini and Agrillo, 2015). We cannot speculate on the exact numerical system used by fish in the present study. However, future studies using operant conditioning with larger numerosities (e.g., 8 vs. 12) are planned to assess whether the difference between lateralized and non-lateralized fish reported here will be also found in the typical ANS range.

One may argue that all our guppies were trained to select the larger group, thus preventing us to assess whether the same result would have been obtained with subjects trained to select the smaller group. With respect to this issue, a previous study using a similar operant conditioning procedure showed no difference in performance when guppies were trained to select the larger or the smaller group (Agrillo et al., 2014). In addition, as stimuli were strictly controlled for non-numerical continuous quantities, lateralized and non-lateralized guppies were required to compare the two groups by using numerical abilities only. Here, there is no reason to expect a differential performance as a function of the numerosity reinforced.

Lateralized guppies could discriminate numerical contrasts that previous studies showed to be close to their numerical acuity threshold: 4 vs. 6 (social companions as stimuli), (social companions as stimuli, Agrillo et al., 2012), and 3 vs. 4 (two-dimensional objects Agrillo et al., 2014). The results of the present study are broadly consistent with comparative studies on a wide range of vertebrate taxa, which suggest that most animals have difficulty making accurate judgments between sets of objects that are closely matched in absolute terms. Studies species ranging from primates, birds, and fish all suggest that sets containing four objects seems to be the upper limit (Hauser et al., 2000; Ward and Smuts, 2007; Hunt et al., 2008; Agrillo et al., 2012). Here, we show that to some extent this limit is determined by the strength of cerebral lateralization, with strongly lateralized individuals being capable of distinguishing between sets of 3 vs. 4 while non-lateralized individuals could only distinguish between 2 vs. 3. This provides further evidence that numerical ability is limited by cognitive capacity and that having a strongly lateralized brain enhances this capacity.

Previous studies on chicks, pigeons and parrots (Güntürkün et al., 2000; Rogers et al., 2004; Magat and Brown, 2009) have all shown that cerebral lateralization can enhance cognitive performance in ecologically relevant contexts. It has been hypothesized that lateralization increase the brain’s capacity to carry out simultaneously different tasks by processing specifics types of information in different areas of the brain. Rogers et al. (2004) observed chicks on a dual task; discriminate food from pebbles while monitoring for an overhead predator. Strongly lateralized chicks detected the model predator sooner and learned to avoid pecking at pebbles faster than the weakly lateralized chicks. Güntürkün et al. (2000) showed that stronger visual asymmetry enhanced the efficiency in discriminating grain from pebbles in pigeons and finally Magat and Brown (2009) examined the influence of lateralization on problem solving (a pebble-discrimination test and a string-pull problem) by Australian parrots and found that strongly lateralized parrots were more efficient than weakly lateralized ones. In some contexts, however, cerebral lateralization can also reduce cognitive performance (Brown and Braithwaite, 2004; Dadda et al., 2009). Thus future studies should try to identify the various costs and benefits associated with laterality within a single model system across multiple domains.

It might be argued that the choice of laterality assay biased the results towards those fish that have a strong inclination to shoal with their mirror image. There are two lines of reasoning that allow us to discount this possibility. Firstly, previous studies have shown strong associations between various tests of laterality in this and other species (Dadda et al., 2012). Thus the outcome would likely to have been the same even if we had examined laterality using an alternative assay (Bisazza et al., 1997). Secondly, we deliberately eliminated any fish that did not spend sufficient time closely associated with its mirror image (close to a quarter of all subjects). Thus it is not the case that non-lateralized fish do not choose to school with their mirror image, but rather they tend to oscillate back and forth between the left and the right eye. All three groups spent roughly 90% of their time close to the mirror.

To conclude, our data provide evidence that numerical discrimination is positively influenced by the strength of laterality and that ubiquitous numerosity mechanisms amongst vertebrates are limited by cognitive capacity. Future studies should attempt to evolutionary and ecological forces that shape selection for numerical competency.

## Author Contributions

All authors (MD, CA, AB and CB) designed the study, set up the procedure, analyzed the data, and wrote the article.

## Conflict of Interest Statement

The authors declare that the research was conducted in the absence of any commercial or financial relationships that could be construed as a potential conflict of interest.
